# RelB regulates the homeostatic proliferation but not the function of Tregs

**DOI:** 10.1186/s12865-020-00366-9

**Published:** 2020-06-18

**Authors:** Shuping Zhou, Weiwei Wu, Zhaoxia Wang, Zhaopeng Wang, Qinghong Su, Xiaofan Li, Yong Yu, Weidong Zhang, Mingzhao Zhu, Wei Lin

**Affiliations:** 1 Institute of Basic Medicine, Shandong Provincial Hospital Affiliated to Shandong First Medical University, Jinan, 250062 China; 2 Key Laboratory of Infection and Immunity, Institute of Biophysics, Chinese Academy of Sciences, Beijing, 100101 China

**Keywords:** RelB, Treg, Cells proliferation, STAT5

## Abstract

**Background:**

RelB, a member of the NF-κB family, plays a critical role in the development of T cells. However, the role of RelB in Foxp3^+^ regulatory T cells (Tregs) remains controversial.

**Results:**

Using a bone marrow chimeric mouse model, we demonstrated that the expansion of Foxp3^+^ Tregs in vivo could be mediated by extrinsic mechanisms. RelB plays an important role in inhibiting the homeostatic proliferation of Tregs, but not their survival. Even with the heightened expansion, *RelB*^−/−^ Treg cells displayed normal suppressive function in vitro. Among the expanded populations of Treg cells, most were nTreg cells; however, the population of iTregs did not increase. Mechanistically, RelB seems to regulate Treg proliferation independently of the signal transducer and activator of transcription 5 (STAT5) pathway.

**Conclusions:**

These data suggest that RelB regulates Treg proliferation independently of the STAT5 pathway, but does not alter the function of Tregs. Further studies are warranted to uncover such mechanisms.

## Background

CD4^+^ CD25^+^regulatory T cells (Treg) are of central importance for the maintenance of peripheral tolerance and the regulation of cellular immune responses. Tregs is divided into two groups based on their origin and phenotypic characteristics: naturally occurring Tregs (nTregs) from the thymus and induced Tregs (iTregs) from the periphery [1–4]. Foxp3 is an important marker [5] and a key transcription factor that regulates the differentiation and function of Tregs. The homeostasis of Treg cells is important for sustaining their function and maintaining the immune balance. Appropriate Treg homeostasis at the periphery plays a crucial role in the maintenance of self-tolerance. Disturbances in this balance are frequently associated with autoimmune diseases. Unlike the homeostasis of naive conventional T cells, Treg homeostasis at the periphery is a much more dynamic process, and its underlying molecular mechanisms has been one of the important topics in this field.

The NF-κB signaling pathways, including the canonical and non-canonical NF-κB signaling pathways, play an important role in the development and maintenance of the peripheral Treg population [6, 7]. RelB is an important transcription factor of non-canonical pathway of NF-κB family that regulates diverse immune and inflammatory responses [8–10]. Previous studies showed that germline deletion of *RelB* caused perturbation in the T cell repertoire, which suggests that RelB is required for T cell development [11–13]. However, the role of RelB in the development of Foxp3^+^ regulatory T cells (Tregs) remains controversial. A study reported that the percentage of Foxp3^+^ Tregs was increased in *RelB* deficient mice, but the absolute number of CD4^+^Foxp3^+^ Tregs is comparable to that of *RelB*^+/−^ mice [14]. By contrast, other studies reported normal T cell development in *RelB*^−/−^ mice [15, 16]. These differences may occur since Treg development is influenced by stromal cells of lymphoid origin, and RelB is involved in the regulation of stromal cells [17–22]. Additionally, Foxp3^+^ Tregs from *RelB* deficient mice up-regulated certain activation markers and effector molecules on the cell surface [20]. Furthermore, the intrinsic role of RelB signaling in regulating the homeostasis and competitive fitness of Tregs was also identified [23]. However, the inhibitory function of RelB on effector T cells is not different from the role of Tregs in wild-type mice [20]. Currently, the role of RelB in the generation and suppressive activities of Foxp3^+^ Tregs is still not clear.

Our study used chimeric mouse models with bone marrow cells from wild-type (WT) or *RelB* deficient mice to study the role of RelB in regulating the proliferation and function of Tregs and their subsets. We also investigated the possible mechanisms of RelB on the proliferation of Tregs to demonstrate the role of RelB in the homeostatic proliferation and function of Treg.

## Results

### RelB influences the frequency of thymic and peripheral Treg cells

**Fig. 1:**
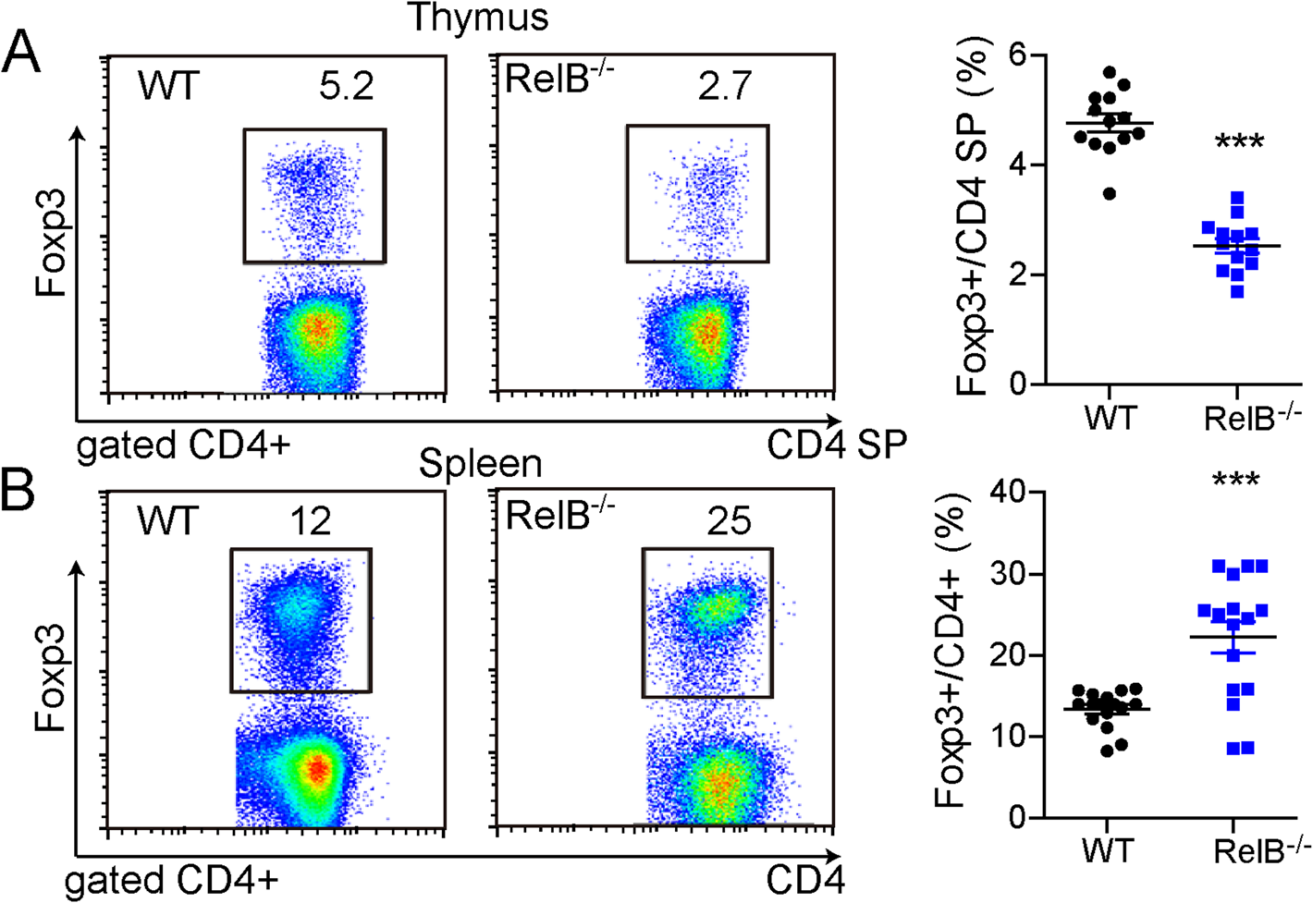
Decreased thymic but increased peripheral Treg cell frequency in *RelB* deficient mice. CD4^+^ T cells were obtained from the thymus and spleen of 6 weeks old WT or *RelB*^−/−^ mice, and were assayed by FACS. **a** Thymic Treg FACS plot and statistic analysis. **b** Splenic Treg FACS plot and statistic analysis. *** *P* < 0.001

To study the role of RelB on the regulation of Tregs, we first measured the percentage of Treg cells in the thymus and spleens of *RelB*^*−/−*^ mice. The percentage of CD4^+^ Foxp3^+^ T cells (Tregs) out of the total population of CD4^+^ T cells was reduced in the *RelB*^*−/−*^ thymus (Fig. 1a). By contrast, the proportion of Tregs out of the total CD4^+^ T cell population in the spleen of *RelB*^*−/−*^ mice was increased (Fig. 1b). Similar to previous reports [19, 24–26], *RelB*^*−/−*^ mice have reduced thymic cellularity and markedly fewer medullary thymic epithelial cells, which provide a delicate microenvironment for the negative selection and induction of Treg cells.

**Fig. 2:**
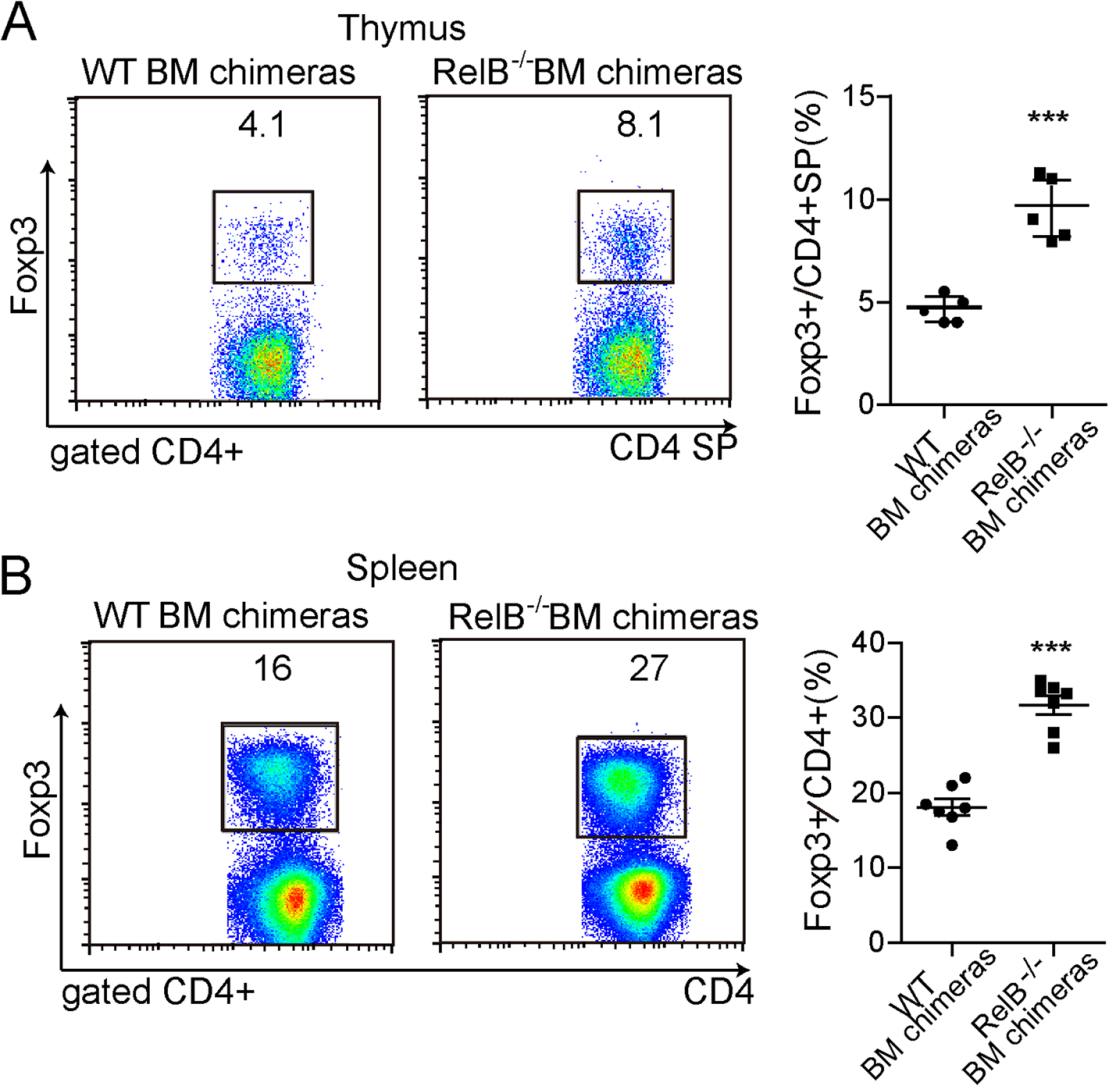
Increased thymic and peripheral Treg cell frequency in *RelB*^−/−^ bone marrow chimeric mice. The bone marrow from 6 weeks old WT or *RelB*^−/−^ mice were transferred to the lethal irradiated mice. 8 weeks later, the mice were assayed for thymic and splenic Treg frequency by FACS. **a** Thymic Treg FACS plot and statistic analysis. **b** Splenic Treg FACS plot and statistic analysis. *** *P* < 0.001

We generated bone marrow chimeric mice to determine the role of RelB in the regulation of Tregs in vivo. Six-week-old CD45.1 mice were irradiated with (Cobalt-60) Co60 at (a dose of 10 Gy). The next day, 5 × 10^6^ donor bone marrow cells from WT or *RelB*^*−/−*^ (CD45.2) mice were intravenously transferred. After 8 to 12 weeks, the percentages of Treg cells from the thymus and spleen of the chimeric mice were analyzed by flow cytometry. The proportion of Tregs in the thymus and spleen of the mice received bone marrow cells from *RelB*^*−/−*^ mice, was higher than those of animals that received bone marrow cells from WT mice (Figs. 2a-b, *P* < 0.001). Excluding the effect of stromal cells and epithelial cells on Tregs, *RelB* deficiency enhanced the proportion of Treg cells in the spleen and thymus.

### RelB controls the development of nTreg in the bone marrow chimeric mouse model

**Fig. 3:**
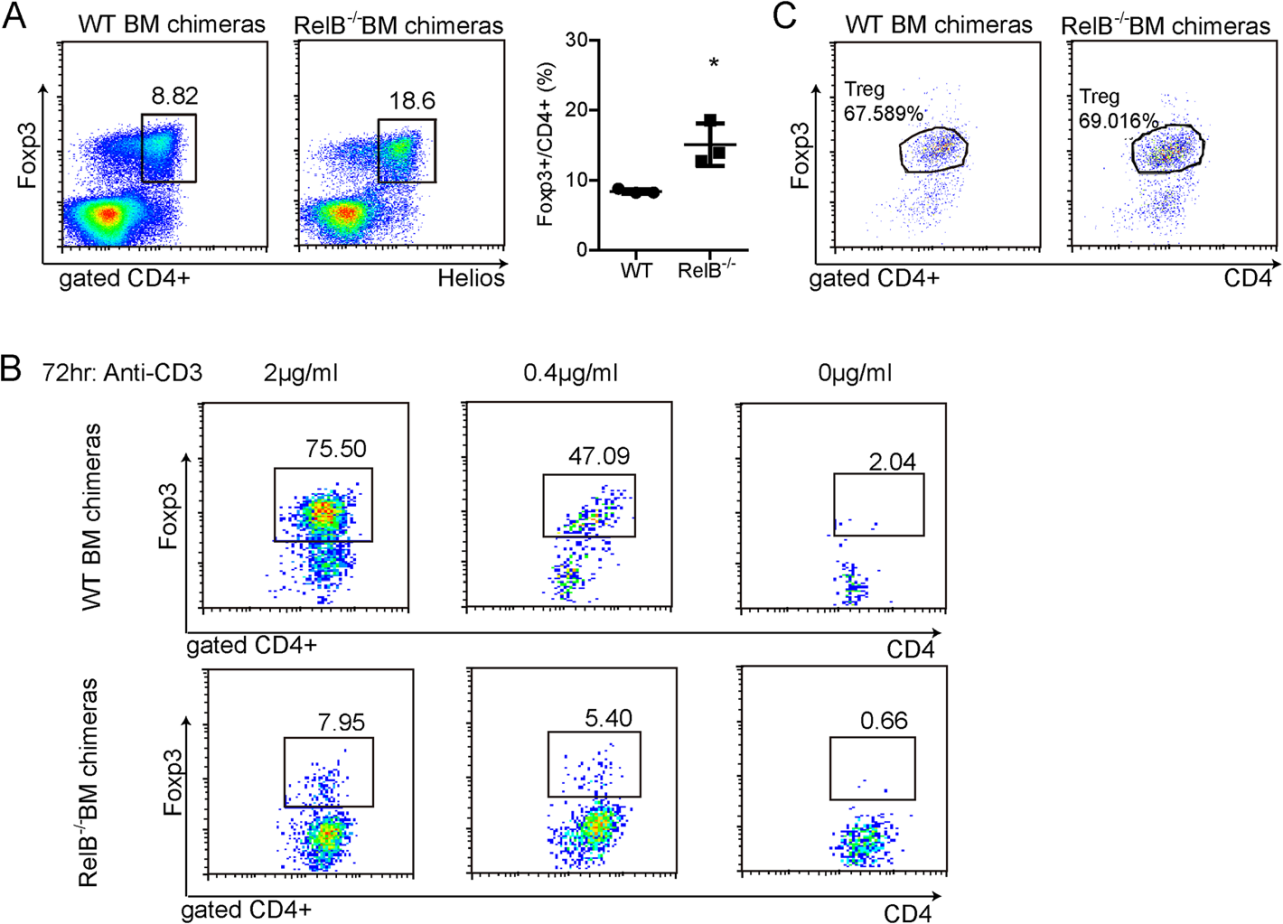
RelB controls nTreg but not iTreg development. The bone marrow from 6 weeks old WT or *RelB*^−/−^ mice were transferred to the lethal irradiated mice. 8 weeks later, the mice were assayed for splenic Treg frequency and helios expression by FACS (**a**) and the naive CD4^+^ T cells were sorted by FACS to induce the iTreg with anti-CD3 antibody for 72 h to detect the Foxp3 expression (**b**). **c** Treg precursor cells (CD4^+^CD8^−^CD25^+^CD69^+^CD24^+^) into mature Treg cells after IL-2 treatment were detected by FACS

Regulatory T cells can be divided into two groups: naturally occurring Tregs (nTregs) from the thymus and induced Tregs (iTregs) from the periphery. To determine which groups of cells in the peripheral Treg were affected by *RelB* deficiency, we further measured the expression of Helios, a marker of nTregs. After bone marrow cells from WT or *RelB*^*−/−*^ mice were transferred to the lethally irradiated mice for 8 weeks, we measured the expression of Helios, and Foxp3 among Tregs by flow cytometry. There was a greater population of FoxP3^+^Helios^+^ Tregs (nTregs) among mice that received bone marrow transplants from *RelB*^*−/−*^ mice compared to those that received a transplant from WT mice (Fig. 3a). At the same time, we also assessed the ability of the naive conventional T cells and the thymic Treg precursor cells from *RelB*^*−/−*^ bone marrow chimeric mice or WT mice to induce iTregs. We found that the naive CD4^+^ T cells from *RelB*^*−/−*^ bone marrow chimeric mice had a lower capability to form Foxp3^+^ Tregs compared to those from WT bone marrow chimeric mice (Fig. 3b). However, the CD4^+^CD8^−^CD25^+^CD69^+^CD24^+^ thymic Treg precursor cells from *RelB*^*−/−*^ bone marrow chimeric mice can induce Foxp3^+^ T cells compared to those from WT bone marrow chimeric mice (Fig. 3c). These results indicated that *RelB* deficiency only influences the development of nTregs in the bone marrow chimeric mouse model. Thus, RelB plays a different role in the regulation of nTregs and iTregs.

### RelB negatively regulates the homeostatic proliferation of Treg by Treg-extrinsic factors

**Fig. 4:**
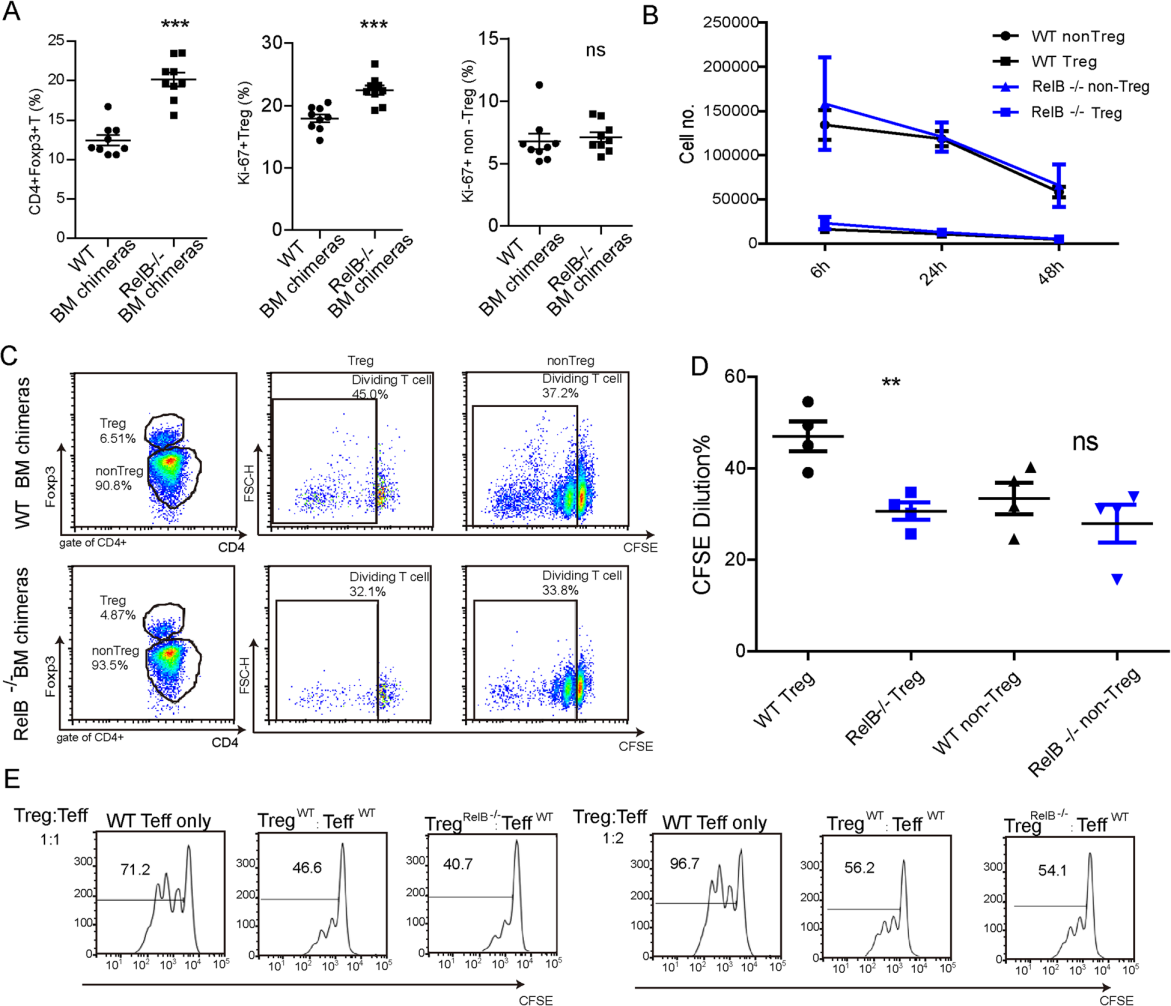
The role of RelB in the proliferation and function of Treg. The single bone marrow chimeras were generated with WT or RelB−/− bone marrow cells. 8 weeks later, **a** the percentage of Treg, Ki67^+^Treg or Ki67^+^CD4^+^Foxp3^−^T cells of WT or *RelB*^−/−^ bone marrow chimeric mice were measured by FACS. *** *P* < 0.001. **b** The splenic WT or *RelB*^−/−^ T cells were sorted by FACS and cultured in vitro. The cell number was measured at 6 h, 24 h and 48 h respectively. **c** the splentic T cells were sorted and labeled with CFSE, and then these cells were transferred to the sublethal irradiated mice. 3.5 days later, the transferred T cells labeled with CFSE were detected by FACS plot and statistic analysis (**d**). **e** CD4^+^CD25^+^ Treg cells from WT or *RelB*^−/−^ mice were isolated and mixed with CFSE labeled naïve conventional CD4^+^ T cells (2 × 10^5^) at indicated ratio. 1 μg/ml anti-CD3/CD28 was used to stimulate T cell proliferation. 3 days later, CD4^+^ T cell proliferation was measured by FACS. ** *P* < 0.01

*RelB* deficiency enhances the frequency of Tregs in the spleen (Fig. 4a, left panel). To determine whether the increased number of Tregs in the spleens of *RelB*^*−/−*^ bone marrow chimeric mice was caused by proliferation, we analyzed the expression of Ki67, a marker of proliferation for CD4^+^ T cells. Our results showed that the population of Ki67^+^ Tregs from *RelB*^*−/−*^ bone marrow chimeric mice was higher than the population from WT bone marrow chimeric mice. However, the percentage of Ki67^+^CD4^+^Foxp3^−^ T cells from *RelB*^*−/−*^ bone marrow chimeric mice was not different from that of WT bone marrow chimeric mice (Fig. 4a). At the same time, we isolated the splenic Tregs from WT and *RelB*^*−/−*^ bone marrow chimeric mice and cultured them in vitro to detect the survival of T cells at 6, 24, 48 h. We analyzed the survival of following populations: WT Tregs, *RelB*^*−/−*^ Treg, WT CD4^+^CD25^−^ T cells (non-Tregs), and *RelB*^*−/−*^ CD4^+^CD25^−^T cells (*RelB*^*−/−*^ non-Tregs) and found no difference in the survival of these T cells (Fig. 4b). *RelB*^*−/−*^ Tregs display normal survival in vitro.

Furthermore, we also measured the rate of CD4^+^ T cell proliferation in vivo. Splenic CD4^+^ T cells from WT or *RelB*^*−/−*^ mice were sorted and labeled with carboxyfluorescein succinimidyl ester (CFSE) respectively, and then were transferred them to the host mice, which were irradiated with a 5 Gy dose of Co60. To our disappointment, Treg cells from *RelB*^*−/−*^ bone marrow chimeric mice showed a decreased rate of proliferation compared with WT bone marrow chimeric mice (Figs. 4c, d). The above results show that the proliferation of *RelB*^*−/−*^ Tregs may be regulated by exogenous factors.

We also want to know if the function of Treg cells from *RelB*^*−/−*^ mice was normal. WT or *RelB*^*−/−*^ Tregs were isolated and mixed with CFSE-labeled naive conventional CD4^+^ T cells (2 × 10^5^) at different ratios. Anti-CD3 (1 μg/mL) and anti-CD28 (1 μg/mL) antibodies were used to stimulate T cell proliferation. Three days later, we measured T cell proliferation using flow cytometry. The results indicated that the WT and *RelB*^*−/−*^ Tregs displayed comparable suppressive function in vitro*,* and RelB did not influence the function of Tregs (Fig. 4e).

### *RelB*^*−/−*^ Tregs did not display higher levels of phosphorylated signal transducer and activator of transcription 5 (STAT5) than WT Tregs in vitro

**Fig. 5:**
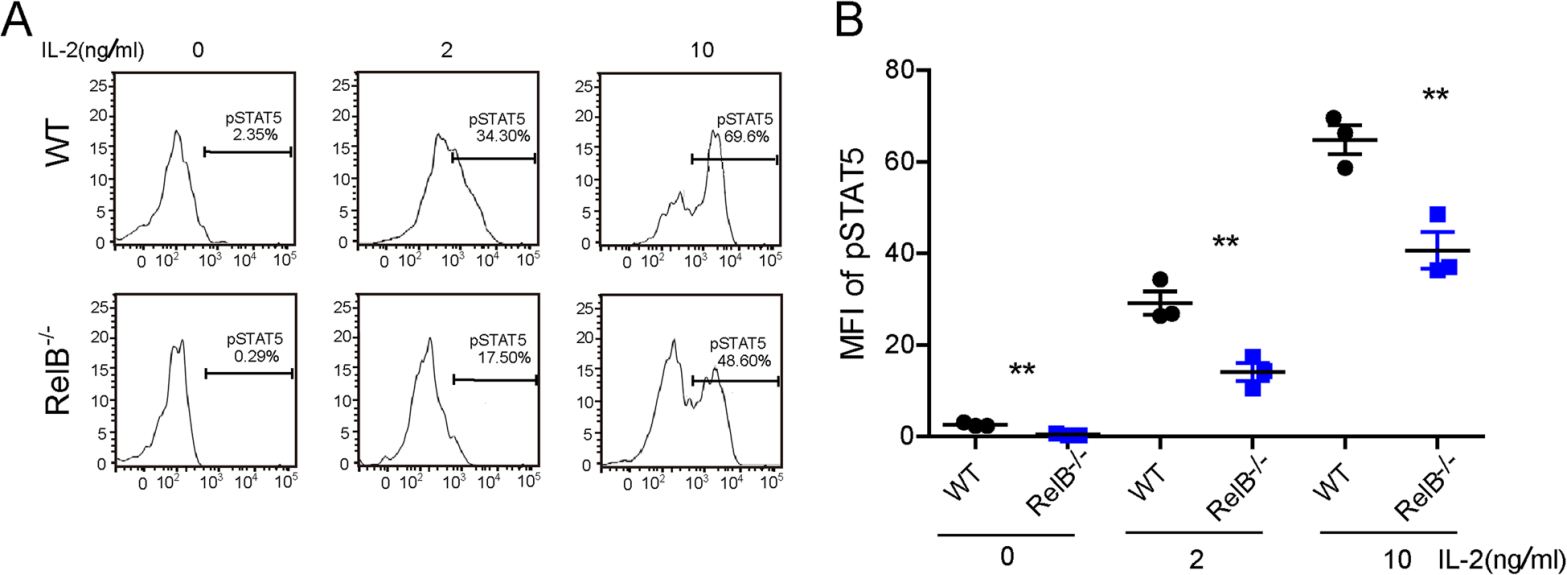
The expression of phosphorylated STAT5 in *RelB*^−/−^ Treg cells. **a** The T cells from *RelB*^−/−^ and WT bone marrow chimeras were isolated and stimulated with indicated concentration of recombinant IL-2 in vitro for 30 min, then the phosphorylation of STAT5 was measured by FACS. **b** Statistic analysis of mean fluorescence index of pSTAT5 at different conditions. ** *P* < 0.01

Signal transducer and activator of transcription 5 (STAT5) is an important transcription factor for maintaining the expression and stability of Treg. The level of phosphorylated STAT5 is correlated with the proliferation of Treg cells. So we measured the levels of phosphorylated STAT5 (pSTAT5) in Tregs from WT or *RelB*^*−/−*^ mice in vitro. Tregs were isolated from *RelB*^*−/−*^ mice and cultured in 24-well plates. After stimulation with different concentrations of IL-2 (0, 2, and 10 ng) for 30 min, the cells were harvested and the level of phosphorylated STAT5 was measured by flow cytometry. We did not detect higher pSTAT5 levels in Tregs from *RelB*^*−/−*^ mice compared to WT mice (Fig. 5). RelB seems to regulate the proliferation of Treg independently of the STAT5 pathway.

## Discussion

In this study, we demonstrated that *RelB* deficiency exerts a profound effect on the proliferation of Foxp3^+^ Tregs, and markedly expands the Treg pool in the periphery. RelB, a component of the NF-κB complex of transcription factors, is a critical regulator of differentiation among medullary thymic epithelial cells and hematopoietic cells [15, 18, 19]. Besides decreased thymic cellularity, *RelB*^*−/−*^ mice had the following abnormal phenotypes: multifocal, mixed inflammatory cell infiltration in several organs, myeloid hyperplasia, splenomegaly due to extramedullary haematopoiesis, and a decreased population of thymic dendritic cells. *RelB* deficiency decreases the population of Tregs in the thymus but increases the thymus Treg populations of bone marrow chimeric mice. These findings indicate that RelB may play a critical role in regulating Treg cell development and homeostasis by influencing the function and status of non-hematopoietic cells. *RelB* deficiency did not influence the proliferation and survival of Tregs directly in our experiments nor other reports [20]. RelB may control the development and proliferation of Tregs through extrinsic cell populations [11, 12, 27–31]. Dendritic cells (DC) and Foxp3^−^ T cells were confirmed to regulate the expansion of Tregs through RelB-dependent secretion of cytokines secretion [32, 33]. The effect of the NF-κB pathway on T cell activation is largely driven through the activation of DC. Thus, the development of Tregs in RelB^*−/−*^ mice was influenced by exogenic factors. Additionally, mice deficient in the non-canonical NF-κB component gene NF-κB 2 (p100), which inhibits RelB activation and participates in RelB nuclear activity, showed normal thymic development and suppressive function of Tregs. However, they had higher populations of peripheral “effector-phenotype” Tregs (eTregs) [23, 34, 35]. This demonstrates that with p100 inhibition of RelB possibly maintaining the suppressive functions of Treg. Therefore, RelB may be a negative regulator but not the master regulator of Treg development.

Furthermore, our study showed the increased Tregs in *RelB*^*−/−*^ bone marrow chimeric mice were primarily nTregs rather than iTregs. These results suggest that RelB may control the homeostasis of nTregs but not iTregs. However, a small proportion of *RelB*^*−/−*^ CD4^+^ T cells were induced to be Foxp3^+^ Tregs compared with WT CD4^+^ T cells. This indicates the role of RelB in induced Foxp3^+^ Treg. Further studies are undoubtedly required to explore the mechanism of RelB on the regulation of nTregs and iTregs.

Additionally, RelB may regulate the transcription of genes involved in the generation of Tregs [20]. Tregs in *RelB*^*−/−*^ mice upregulated certain activation markers and effector molecules on the cell surface, including CTLA-4, KLRG1, and TIGIT [20]. Our research and other studies [14, 20, 33] have found that *RelB*^*−/−*^ Tregs showed similar suppressive activities as their WT counterparts. The deletion of RelB did not influence the proliferation of Tregs in vitro, but increased the proliferation of Tregs in vivo. These data suggest that the role of RelB in the function and proliferation of FoxP3^+^ Tregs. Therefore, the molecular mechanismof RelB on Tregs remain to be studied in the future.

Furthermore, we found that with IL-2 stimulation, the levels of pSTAT5 in Tregs from *RelB*^*−/−*^ mice were not higher than those from WT Treg. RelB deficiency by itself does not affect the Treg functions. Previous reports had shown that the expansion of Foxp3^+^ Tregs in *RelB*^*−/−*^ mice were mediated primarily by the hyperactivation of FoxP3^−^ T effector cells that spontaneously produce increased levels of IL-2, a growth factor for Foxp3 ^+^ Tregs [18, 19, 36–39]. IL-2 is an activator of STAT5 signaling [40]. Upon IL-2 stimulation, RelB seems to regulate Treg proliferation independently of the STAT5 pathway. *RelB*^*−/−*^ Treg cells may have a weaker response to IL-2 than WT Treg cells. Whether IL-2 promotes the proliferation of *RelB*^*−/−*^ Tregs through other factors or pathways requires further investigation.

## Conclusions

Our study identified a critical role for RelB in regulating the homeostasis of Foxp3^+^ Tregs. This effect was shown to be mediated by Treg cell-extrinsic mechanisms and occurred independently of STAT5 signaling. The possible endogenous factors that regulate the proliferation of Treg in *RelB*^*−/−*^ mice need to be identified in future studies. Furthermore, the importance and complexity of RelB in the regulation of the immune system to ensure homeostasis and immune tolerance require further study.

## Methods

### Animals

*RelB*^*−/−*^ mice and CD45.1 mice (6 weeks of age, female) were gifts from Dr. Y. X. Fu (University of Texas Southwestern Medical Center, Dallas, TX, USA). WT C57BL/6 mice (6 weeks of age, female) were purchased from Vital River Laboratory Animal Technology (Beijing, China). All mice were housed under specific pathogen-free conditions in the laboratory animal room of Institute of Biophysics, Chinese Academy of Sciences. All animals were housed with a 12 h light/dark cycle on ventilated racks with corncob bedding. The cage temperature was maintained from 68 to 76 degrees Fahrenheit. Five animals were housed in each cage. Animals were fed and given water every day. All procedures were performed in compliance with guidelines for the care and use of laboratory animals and were approved by the ethics committee of the Institute of Biophysics, Chinese Academy of Sciences (Beijing, China) and Shandong Academy of Medical Sciences (Shandong, China).

### Bone marrow chimeric construction

Six-week-old CD45.1 mice were irradiated with a 10 Gy dose of Co60. The next day, WT or *RelB*^*−/−*^ (CD45.2) mice were euthanized and bone marrow cells were extracted from the thigh bone to form a single-cell suspension. Donor cells (5 × 10^6^) were intravenously transferred to recipients. Mice were continuously fed sulfamethoxazole and trimethoprim (Bactrim) for 4 weeks starting 1 day before irradiation. Five mice per group were used for experiments. Six to 8 weeks later, mice were euthanized by CO_2_ inhalation followed by cervical dislocation for thymocyte and splenocyte analysis.

### Flow cytometry and antibodies

Mouse splenocytes and thymocytes were prepared from pooled thymus or spleen. Thymic and splenic cells were pre-incubated with Fc-block before staining with other antibodies. The mouse antibodies used included anti-CD4 (RM4–5, eBioscience); anti-CD8 (53–6.7, eBioscience); anti-CD45.1 (A20, BioLegend), anti-CD45.2 (104, eBioscience); anti-CD25 (PC61.5, eBioscience); anti-CD69 (H1.2F3, eBioscience); anti-CD24 (M1/69, eBioscience); anti-Helios (22F6, eBioscience) before flow cytometry analysis. For intracellular staining of Ki-67 (B56, BD), pSTAT5 (47/Stat5(pY694), BD) and Foxp3 (NRRF-30, eBioscience), cells were fixed and permeabilized with BD Cytofix/Cytoperm™ Fixation / Permeabilization Solution Kit (554,714, BD) and stained according to the manufacturer’s protocols. The CD4^+^ T cells from the thymus and spleen of WT or *RelB*^*−/−*^ mice were analyzed by flow cytometry and gated as shown in the Supplementary Figure 1. The samples were analyzed using a BD LSRFortessa flow cytometer and FlowJo software (Tree Star Inc). All single-cell suspensions from the tissues were stained with Abs diluted in PBS containing 2% FCS for 30 min on ice.

### Adoptive cell-transfer experiments

The WT and *RelB*^*−/−*^ splenic CD4^+^ T cells were labeled with 5.0 μM 5- (and 6-) CFSE (Molecular Probes, Inc. Eugene, OR, USA) for 15 min at 37 °C. Then the cells were washed with PBS twice before re-suspending in PBS. For adoptive transfer, the CD4^+^ T cells (CD45.1^+^CD4^+^) derived from WT or (CD45.2^+^CD4^+^) derived from *RelB*^*−/−*^ mice were respectively transferred into CD45.1^+^ CD45.2^+^ WT B6 mice intravenously. At 3.5 days after cell transfer, the phenotype of transferred CD45.1^+^ CD4^+^ T cells or CD45.2^+^ CD4^+^ T cells in the host spleen was assessed by flow cytometry by gating on live CD4^+^ T cells.

### The induction of iTregs from the spleen

Naive CD4^+^ CD25^−^ T cells from WT or *RelB*^*−/−*^ splenocytes were sorted using a BD Aria III flow cytometer (BD Biosciences, CA, USA). The purity of CD4^+^CD25^−^ T cells was routinely above 90%, and these cells were cultured in 96-well plates with TGF-β (5 ng/mL; Peprotech) and IL-2 (50 U/mL; R&D) stimulation. Three days later, flow cytometry was used to measure the expression of CD4^+^ Foxp3^+^ T cell (iTreg).

### Isolation and induction of Treg precursor cells

Treg precursors were defined as CD4^+^CD8^−^CD25^+^CD69^+^CD24^+^ cells that were isolated from the thymocytes of WT or *RelB*^*−/−*^ mice and sorted using a BD Aria III flow cytometer (BD Biosciences, CA, USA). The purity of Treg precursor cells was routinely above 90%. Cells were harvested and stimulated with IL-2 (50 U/mL; R&D) for 3 days to induce the Treg phenotype. Single-cell suspensions were collected, stained, and detected using flow cytometry.

### The inhibition of Treg on T cells

WT or *RelB*^*−/−*^ CD4^+^ CD25^+^ Treg cells were isolated and mixed with CFSE-labeled naive conventional CD4^+^ T cells (2 × 10^5^) at different ratios (Treg: conventional T = 1:1, 1:2). Anti-CD3 (1 μg/mL) and anti-CD28 (1 μg /mL) antibodies were used to stimulate T cell proliferation. Three days later, T cell proliferation was measured by flow cytometry.

### Statistical analysis

Flow cytometry data were analyzed with FlowJo (Tree Star) software. Numerical data were processed in Excel (Microsoft) and plotted in Graphpad Prism (Graphpad Software, Inc). Statistical significance was determined using the nonparametric Mann–Whitney U test. *P < 0.05, **P < 0.01, and ***P < 0.001 unless otherwise indicated.

## Supplementary information

**Additional file 1: Supplementary Figure 1.** The gate of CD4^+^T cells in the thymus (A) or spleen (B) of the WT mice and RelB deficient mice.

## Data Availability

All data is available upon author request.

## Abbreviations

Treg: Regulatory T cells
nTreg: Natural regulatory T cells
tTreg: Thymic regulatory T cells
pTreg: Peripherally regulatory T cells
iTreg: Induced regulatory T cells
pSTAT5: Phosphorylation activator of transcription 5
NF-κB: Nuclear factor kappaB
CFSE: Carboxyfluorescein diacetate succinimidyl ester
WT: Wild-type
CTLA-4: Cytotoxic T-lymphocyte-associated protein 4
KLRG1: Killer cell lectin-like receptor subfamily G member 1
TIGIT: T-cell immunoglobulin and immune-receptor tyrosine-based inhibitory motif (ITIM) domain
Foxp3: Forkhead box P3
FCS: Fetal calf serum
FACS: Fluorescence-activated cell sorting.
