# Supplementary material for: RelB regulates the homeostatic proliferation but not the function of Tregs
Source: BMC Immunol. 2020 Jun 18;21:37. doi: 10.1186/s12865-020-00366-9 (PMC7302365; doi:10.1186/s12865-020-00366-9)
Supplement: Supplementary file 1 — Additional file 1: Supplementary Figure 1. The gate of CD4+T cells in the thymus (A) or spleen (B) of the WT mice and RelB deficient mice. [file 12865_2020_366_MOESM1_ESM.docx]

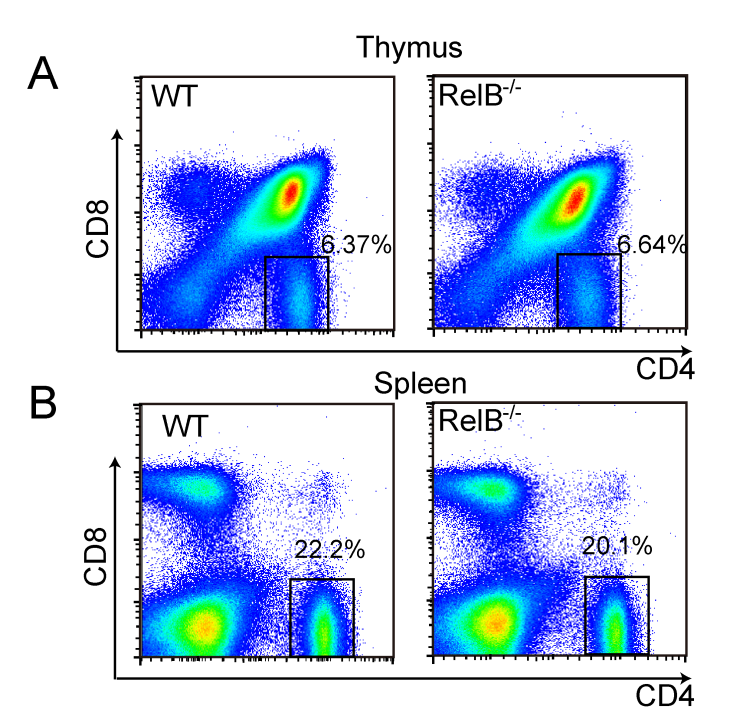


**Supplementary Figure 1. The gate of CD4^+^T cells in the thymus (A) or spleen (B) of the WT mice and RelB deficient mice.**
